# Thymomas With Intravascular and Intracardiac Growth

**DOI:** 10.3389/fonc.2022.881553

**Published:** 2022-06-24

**Authors:** Andrea Valeria Arrossi, Josephine K. Dermawan, Michael Bolen, Daniel Raymond

**Affiliations:** ^1^ Department of Pathology, Robert J. (R.J) Tomsich Pathology and Laboratory Medicine Institute, Cleveland Clinic, Cleveland, OH, United States; ^2^ Department of Pathology, Memorial Sloan Kettering Cancer Center, New York, NY, United States; ^3^ Imaging Institute, Cardiovascular and Thoracic Radiology, Cleveland Clinic, Cleveland, OH, United States; ^4^ Department of Thoracic Surgery, Cleveland Clinic, Cleveland, OH, United States

**Keywords:** invasive thymoma, staging, intravascular growth, superior vena cava syndrome, intravascular growth pattern

## Abstract

Thymomas are derived from the epithelial component of the thymus and constitute the most common tumor of the anterior mediastinum. These neoplasms are considered malignant for their potential for invasion and metastases. Several histopathologic subclassification schemes have been proposed over the years, however, correlation of histotypes with prognosis remains controversial. In contrast, studies invariably have shown that staging and resection status correlate with oncologic behavior and disease outcomes. In this regard, several staging systems have been presented, though transcapsular invasion and degree of involvement of adjacent anatomic structures are common denominators of all schemes. Involvement of the great vessels and heart most commonly results from direct invasion, which may lead to unusual clinical presentations such as superior vena cava syndrome. Moreover, intravascular and intracardiac growth with or without direct mural invasion rarely occurs. We provide an overview of thymomas with intravascular and intracardiac involvement.

## Introduction

Thymomas are malignant neoplasms derived from the epithelial component of the thymus. While they constitute the most common malignancy primary to the mediastinum, thymomas are rare, with a reported age standardized rate of 0.15 to 0.19/100,000 (1).

Given the complex histologic morphology and architecture of the normal thymus, thymomas are histologically characterized by their morphologic heterogeneity and have remained difficult to categorize by conventional histologic findings. Similarly, while treatment modalities, oncologic behavior, and disease outcomes are delineated by the clinical and pathologic staging, this parameter did not escape from the thymoma controversies in the literature.

Notably, disparity and lack of granularity amongst the current staging systems creates some challenges in the studies of locally advanced thymomas with vascular or cardiac involvement. In the Masaoka-Koga staging system (2) these tumors would classify as stage III. However, there is no differentiation in stage based on organ involvement type thus innominate vein (InV) involvement (often resectable) and direct myocardial involvement (often unresectable) are staged similarly (**Table 1**). The latest version of the TNM staging system does provide greater clarity. Macroscopic invasion into neighboring organs has been further differentiated in the T classification (3). The T3 group includes invasion into adjacent structures that are typically considered resectable and include InV, superior vena cava (SVC), chest wall, phrenic nerve, and extracardiac pulmonary vessels. On the other hand, T4 includes invasion into structures that are classically not considered resectable and include aorta, arch vessels, main pulmonary artery, myocardium, trachea or esophagus (**Table 2**). However, with advances in cardiac and great vessel surgery, paradigms are evolving with respect to determination of resectability. For example, focal great vessel involvement is no longer necessarily considered a formidable challenge.

**Table 1:** Masaoka-Koga Stage.

**I**	Grossly and microscopically completely encapsulated
**II**	a. Microscopic transcapsular invasion b. Macroscopic capsular invasion into thymic or surrounding fatty tissue or grossly adherent to but not breaking through, mediastinal pleura or pericardium
**III**	Macroscopic invasion of neighboring organs pericardium, great vessels, or lung
**IV**	a. Pleural or pericardial dissemination b. Lymphatic or hematogenous metastasis

**Table 2:** TNM Stage.

**T1**	a. Encapsulated or unencapsulated with or without extension into mediastinal fat b. Extension into mediastinal pleura
**T2**	Pericardium
**T3**	Lung, InV, SVC, chest wall, phrenic nerve, hilar extrapericardial pulmonary vessels
**T4**	Aorta, arch vessels, main pulmonary artery, myocardium, trachea, or esophagus

InV, Innominate vein; SVC, Superior vena cava.

**Figure 1:**
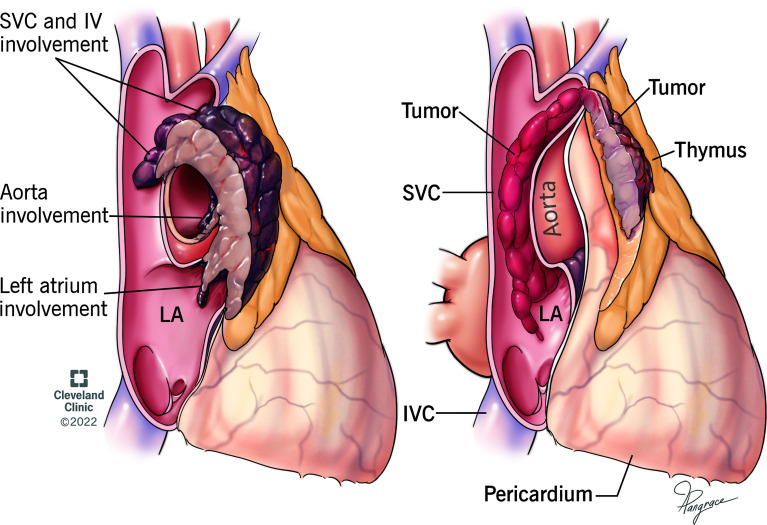
Drawing of 2 different types of vascular invasion. Left: Thymoma invades superior vena cava (SVC), innominate vein (IV), aorta, and left atrium (LA) by contiguous infiltration. Right: Thymoma shows a single point of wall invasion into the vessel with intravascular downstream extension into SVC and LA without contiguous involvement.

Invasion of the great vessels, particularly InV and SVC, is not uncommon in advanced thymomas, however, in most cases, stage III (Masaoka-Koga) or T3 (TNM system) result from invasion of extravascular structures, i.e. lung and/or pericardium (4–8). Furthermore, 2 patterns of vascular invasion may be found. Vessels may be involved by contiguous extension (**Figure 1** Left), or, rarely, by downstream endoluminal growth into the large vessels and heart (**Figure 1** Right).

In this report, we summarize the cases in the English medical literature that provide detail of thymic tumors with intravascular/thrombotic growth protruding into the SVC, its tributaries, and heart, without evidence of contiguous extension through their walls. Our purpose is to highlight this unusual pattern of vascular invasion and generate the base for the identification and further evaluation of these cases. 

## Design

A search of thymomas with vascular involvement was performed in the PubMed database (National Medical Library) using the following search terms: “thymoma” and “vascular” and/or each of the terms “intravascular”, and/or “superior vena cava”, “innominate vein”, “brachiocephalic vein”, “atrium”, “cardiac”, “heart”, “intracardiac”, “thrombosis”.

All pertinent articles in the English language medical literature were reviewed. Only those studies with confirmed endovascular growth as the pattern of vascular involvement (**Figure 1** Left) were included. Articles referring to cases with vascular involvement resulting from adjacent contiguous invasion, or without detailed data regarding the pattern of vascular invasion were excluded (4–7, 9–21).

The clinical, radiographic, and surgical data were gathered and tabulated for each publication.

## Results

Thirty-five publications of patients with confirmed radiographic and/or surgical description of intravascular/intracardiac spread of thymoma without evidence of direct connection were found after thorough review of the numerous articles from the English medical literature. Each publication reported 1 patient with these characteristics (22–55).

The detailed results are shown in **Table 3**. In summary, there were a total of 34 patients (17 females and 15 males, gender not available in 2 cases) with an average age of 58 years old. Twenty-six cases (87%) presented with swelling of the veins of the face, neck, upper extremities, and/or chest wall, characteristic of SVC syndrome, 7 of them associated with dyspnea. SVC syndrome was not present in 5 (17%) patients, 1 was an incidental finding during cardiac surgery, and 4 presented with dizziness and cough, abdominal pain and distension, ptosis, pain and weight loss, ptosis, and chest discomfort respectively. The patient with ptosis was the only one with evidence of myasthenia gravis. Clinical presentation was not provided in 4 patients.

**Table 3:** Patient characteristics and tumor details.

Year	Ref	Age	Gender	Clinical presentation (as described)	MG	Imaging studies	Imaging studies: vessels involved	Intraoperative vessels involved	Imaging: other organs	Intraoperative: oher organs	Largest size (cm)	Histologic type
**1978**	22	70	F	dyspnea, face swelling, hoarseness	No	SVC cavogram	InV, SVC	InV, SVC	N/A	none	16	LE
**1979**	23	39	M	SVC syndrome	No	angiography	SVC	SVC	none	none	N/A	N/A
**1989**	24	80	F	swollen neck veins, pleural effusion	No	echocardiography, angiography	SVC, RA	surgery not performed	N/A	N/A	N/A	spindle cell
**1990**	25	38	M	abdominal pain, weight loss	No	echocardiography	SVC, RA, RV	SVC, RA, RV	none	pericardium	9	N/A
**1990**	26	50	M	Neck and chest venous distension, face swelling	No	CT, SVC cavogram	SVC	SVC	N/A	N/A	10	EP
**1992**	28	65	f	dizziness, cough, anorexia	No	echocardiography, transesophageal US	SVC, RA	SVC, RA	none	none	N/A	N/A
**1992**	27	72	M	swelling of the face and exertional dyspnea	No	CT, SVC cavogram	SVC	InV, SVC, RA	pericardial efussion	pericardium, RUL, LUL	15.5	EP
**1993**	29	56	M	face and upper extremity edema	No	US, CT, MRI, SVC cavogram	SVC, RA	SVC, RA	N/A	pericardium	4	EP
**1994**	39	37	M	SOB, edema upper limb, hemoptysis, cardiac tamponade	No	CT, echocardiogram	SVC	SVC	pericardium, RML, chest wall	pericardium, RML, chest wall	N/A	N/A
**1997**	31	52	F	neck swelling	No	transthoracic 2-dimensional echocardiography	InV, SVC, RA	InV, SVC, RA	none	none	15	N/A
**1999**	32	74	M	eyelid ptosis, myasthenia gravis	Yes	CT	N/A	InV	none	sternothyroid and sternohyoid muscles	5.2	EP
**1999**	33	44	M	facial, upper extremity edema, hoarseness,	No	CT, echocardiogram, venography, gallium scintigraphy	InV, SVC, RA	InV, azygous ven, SVC, RA	none	pericardium, LUL, RUL, RML, right phrenic nerve	N/A	N/A
**2004**	34	64	N/A	anterior chest discomfort	No	CT	InV, SVC	InV, SVC	pericardial effusion	pericardium, RUL	16	B2
**2006**	35	56	F	swelling of face and upper extremities	No	CT, MRI, echocardiogram	InV, SVC, RA	InV, SVC, RA	N/A	LUL, tumor implant in diaphragm	N/A	AB
**2007**	36	71	F	lethargy, facial edema	No	CT, MRI	InV, SVC	InV, SVC	none	left phrenic nerve	6	AB
**2007**	37	48	M	face and the right upper extremity	No	CT, echocardiography, venocavogram	InV, SVC and RA	InV, SVC, LBCV, RA	none	pericardium, RUL, RML, right phrenic nerve	N/A	AB
**2007**	38	86	M	N/A	N/A	MDCT angiography	InV, SVC, RA	no surgery	N/A	N/A	N/A	N/A
**2008**	39	50	F	dyspnea, enlargement of chest vessels	No	CT, cardiac MRI	SVC, RA	N/A	N/A	right side of the sternum.	13	N/A
**2009**	40	53	M	face and upper extremity edema.	No	CT	InV, SVC, right atrium	InV, SVC, RA, tricuspid valve, RV	none	right lung hilum, right phrenic nerve, pericardium	10	AB
**2010**	41	40	N/A	swelling of the face and the left upper extremity	No	CT, echocardiogram	InV, SVC, RA	InV, SVC, RA	pericardial effusion	none	14	B3
**2012**	42	53	F	SVC syndrome	No	CT, echocardiography	SVC, RA	InV, SVC, RA	N/A	right phrenic nerve	8	N/A
**2012**	43	69	M	swelling of the face and bilateral upper extremities	No	CT, MRI	InV, SVC, jugular and subclavian veins	InV, SVC, jugular, subclavian veins	N/A	pericardium	N/A	AB
**2013**	44	44	F	facial and left upper limb edema	No	CT, PET CT, echocardiogram	InV, SVC, RA	InV, SVC, RA	N/A	pericardium	5.2	B3
**2013**	46	39	F	facial edema and dyspnea	No	CT, PET-CT	InV, SVC and right atrium	ImV, SVC, RA	none	none	N/A	B2
**2013**	47	53	F	severe dyspnea and facial oedema	No	NCCT, CCT, MRI	InV, SVC, RA	no data	N/A	N/A	N/A	B3
**2013**	45	74	M	SVC syndrome	No	CT, MRI, angiography	InV, SVC, RA	no surgery	N/A	N/A	6	B2
**2014**	48	54	M	N/A	N/A	CT, MRI	SVC, azygos vein, RA	InV, SVC, azygos vein, RA	N/A	none	3	B3
**2016**	49	57	F	facial swelling	No	CT, CT angiography	InV, SVC	InV, SVC	N/A	N/A	3.5	B2
**2016**	50	74	F	Face and upper extremity swelling, chest wall, jugular veins distention	No	echocardiogram, cardiovascular MRI	SVC, RA	InV, SVC, RA	none	RUL, pericardium, right phrenic nerve	9.9	B1
**2018**	51	84	F	N/A	No	CT, PET/CT	none seen	InV, *via* thymic vein	metastases bilateral lungs		4.4	A
**2019**	52	50	F	N/A	No	CT, echocardiogram	InV, SVC, RA	InV, SVC, RA	pericardial effusion	IVC	5	B3
**2019**	53	39	F	facial and upper limb swelling	No	CT, MRI	InV	InV, SVC	pericardium	lung, pericardium	7.9	B1
**2020**	55	63	F	exertional dyspnea, and upper limb and facial edema	No	N/A	N/A	InV, SVC	none	mediastinal pleura, pericardium, RUL, LUL	12	B2
**2020**	54	76	M	incidental, cardiac surgery	No	CT, MRI	InV, SVC, RA	N/A		N/A	6.5	A

CT, Computed tomography; EP, Epithelial predominant; InV, Innominate vein; LE, lymphoepithelial; LUL, Left upper lobe; MG, Myasthenia gravis; MRI, Magnetic resonance studies; PET, Positron emission tomography; RA, Right atrium; RML, Right middle lobe; RUL, right upper lobe; RV, Right ventricle; SVC, Superior vena cava.

N/A, not applicable.

Patients received a wide range of diagnostic imaging studies, with all but 5 having either magnetic resonance imaging (MRI) and/or computed tomography (CT) imaging performed as part of the evaluation. All 5 patients who did not undergo CT or MRI were evaluated in 1997 or earlier and had echocardiography or catheter-based venography. By imaging studies, invasion of the SVC was seen in 28 (85%) patients, 21 of whom with involvement of the right atrium (RA) and 15 with involvement of SVC tributaries, mostly InV. Vascular invasion was not detected radiographically in 1 patient. Radiographic data was not available in 2 patients. Both intraoperative and imaging data were provided in 27 cases. Of those, concordant findings were present in 20 cases and in 7 cases, 1 or more involved vessels were encountered intraoperatively.

The average size of the thymomas (available in 23 cases) was 8.9 cm, ranging from 3 to 16 cm. The histologic type was reported in 27 cases: WHO types A in 3, AB in 5, B1 in 2, and B2 and B3 in 5. Two cases were reported as epithelial-predominant, ‘mixed” in 1, “type II” in 1, and lymphoepithelial in 1.

## Discussion

Thymomas are the most common primary tumor occurring in the anterior mediastinum, with an annual incidence 0.15 to 0.19/100,000 (1). All age groups are affected, but most commonly they occur in middle-aged adults (40-50 years). Patients may present with symptoms due to mass effect, autoimmune or paraneoplastic syndromes or metastases, and in a subset of patients, thymomas are found incidentally during thoracic imaging. Autoimmune or paraneoplastic symptoms are most commonly, but are not limited to, neuromuscular disorders (myasthenia gravis), immunodeficiency disorders (hypogammaglobulinemia), or hematologic diseases (pure cell aplasia, hemolytic anemia). 

Once a diagnosis is made, a multidisciplinary treatment approach with clinicians experienced with thymoma/thymic carcinoma is vital. Tumor type, stage, extent of invasiveness, potential phrenic nerve involvement, and the physiologic status of the patient are all essential considerations when determining the appropriate multi-modal treatment plan. In the circumstance of potential vascular or cardiac involvement, surgery may still play a role in the patient management depending on degree and location of involvement.

At the time of presentation, most tumors (65%) are Masaoka-Koga stage I or II, 25% are stage III, and around 10% are stage IV (8). Half of all presentations have invasion into surrounding structures but remain candidates for multimodal therapy including surgical resection. This emphasizes the importance of multidisciplinary management. Unfortunately, the Masaoka-Koga staging system makes no differentiation in stage based on organ involvement type thus innominate vein involvement (often resectable) and direct myocardial involvement (often unresectable) are staged similarly (**Table 1**). This is better reflected in the latest version of the TNM staging system, in which invasion of mediastinal organs is further divided into T3 (invasion or lung, InV, SVC, phrenic nerve, chest wall or extrapericardial pulmonary arteries) and T4 (invasion of aorta, arch vessels, intrapericardial pulmonary artery, myocardium, trachea and/or esophagus) (**Table 2**) (3).

We present a review of thymoma cases reported in the English medical literature that demonstrated vascular invasion through endovascular and intracardiac growth not associated with contiguous extension from the main mass (**Figure 1**). SVC syndrome was the most common presentation of these patients. In most cases, imaging studies were able to demonstrate endovascular growth with good correlation with the intraoperative findings. These cases are staged as Masaoka-Koga III or TNM system T3, however they have an unusual unifying component, the intravascular extension of tumor. It is hypothesized that thymomas enter the great veins through small vessels, such as the thymic veins, or focal transmural invasion, analogous to other angioinvasive malignancies such as renal cell carcinoma, leading to its growth along the venous stream down into the larger veins and atrium. Protrusion of the tumor into the thymic veins was noted intraoperatively in some of the cases in this review.

The WHO classification is currently the primary scheme used for the histologic typification of thymomas (56). Types A, AB, B1, B2, and B3 are divided based on cell morphology and progressive loss of the background population of immature thymic lymphocytes. While the prognosis of thymomas is mainly dependent on stage and resection status, Weiss et al. showed that recurrence rate was correlated with the WHO histotypes but not overall survival (57). However, the relevance of the pathologic classification as a prognostic indicator for recurrence and overall survival has been the subject of numerous studies, with discrepant results. Furthermore, several other histologic schemes have been proposed in the literature, and the controversy regarding which one is the most reproducible, applicable to, and reflective of clinical behavior is ongoing.

Histologic data was available in 25 cases and included 14 cases B2 and B3 (3 older cases classified as epithelial predominant were converted to B3 in this review). Surprisingly, types A, AB, and B1 grouped together were reported at frequencies comparable to types B2 and B3.

Imaging plays a central role in the diagnosis and staging of thymomas. Thymomas typically present in an anterior mediastinal location, arising from one side of the thymus with well-defined margins, smooth or lobulated contours, and locations varying from thoracic inlet to cardiophrenic angle (58). Use of intravenous contrast is indicated whenever feasible, as this allows for improved assessment of vascular involvement, as well as enhancement characterization of the mass. Vascular invasion is suggested by alteration of vessel lumen contour, encasement or obliteration of vessel, or soft tissue intravascular extension, which may also extend to pericardium or cardiac chambers (59). Use of ECG synchronized imaging, either by CT or MRI may allow for improved delineation of cardiac involvement.

Involvement of the mediastinal vessels, in particular InV and/or SVC, is not uncommon in locally advanced thymomas (about 15%), however, lung and pericardium are the most frequently involved organs in Masaoka-Koga stage III or TNM T3 tumors (4–8). The impact on tumor behavior of vascular involvement remains unclear, however, recurrence rates tend to be higher, and disease-free survival shorter, in cases with invasion of the great vessels compared to those without (4, 6, 7). More so, almost no studies evaluate the significance of differentiating specific vessel involvement. In this regard, in a study of clinicopathologic correlation of 250 thymoma cases by Moran et al. the InV was stratified separately from the other great vessels and heart as stage IIA and stage IIC respectively. This stratification did not result statistically significant, however, the number of cases in the study was low, with only 56 tumors stage IIA and 2 tumors stage IIC. Nonetheless, these groups were still included in the proposed staging system. The authors manifest that such stratification is important for the possibility of advances in surgical techniques, additional therapy, and future larger studies. (**Table 4**) (60). For instance, extended involvement of the surrounding anatomic structures (Masaoka stage III or TNM system T3) makes radical surgery unfeasible in up to 30% to 40% of invasive thymomas and thymic carcinomas grouped together (20). Involvement of the InV, however, can be addressed with simple vein resection with or without reconstruction, and involvement of the SVC can be addressed surgically in certain circumstances although cardiopulmonary bypass may be necessary. Thus, there is likely significantly variability in the determination of resectability based on surgeon and center level experience.

**Table 4:** Moran Stage.

0	Encapsulated tumor
I	Invasive tumor into perithymic adipose tissue
II	Direct invasion
	A. InV, mediastinal pleura, lung B. Pericardium C. Great vessels (aorta, SVC), heart
III	Metastatic disease
	A. Intrathoracic structures, diaphragm, LNs B. Extrathoracic invasion

InV, Innominate vein; SVC, Superior vena cava; LNs, Lymph nodes.

Notably, Moran et al. also recognized that vascular invasion in thymomas may follow 2 different patterns, either direct wrapping/extension into the vascular wall, or spread within the vessel itself, as the cases reviewed in this article. Whether staging should be different for these tumors is uncertain. The significance of specific vessel differentiation in the stratification of staging systems, and the pattern of vascular involvement would need to be elucidated.

### Limitations

In this study, we present cases of thymoma with intravascular/thrombotic pattern of vascular invasion based on a review of the literature. We excluded the cases that presented vascular invasion by contiguity, as well as those we deemed confusing or ambiguous. However, we recognize that our study is based on a retrospective literature review, and as such, accuracy of the data might be difficult to assess in some cases, especially older reports, which constitutes the main limitation of our study. Nonetheless, we believe that our study could set the base for the identification of these cases and development of future studies to delineate their significance.

## Conclusion 

We provide a descriptive analysis of thymoma cases with vascular invasion resulting from downstream polypoid and/or thrombotic intravascular growth detected with imaging studies and/or intraoperatively. intravascular spread is rare among thymomas, regardless of histologic type or staging, and may create uncertainties regarding management. We acknowledge that precise assessment of the incidence of this phenomenon is challenging due to the ambiguity in defining the pattern of vascular invasion in most studies. Importantly, vital to the most appropriate intra-operative planning and perioperative support in managing patients with thymomas is an ongoing, multidisciplinary evaluation, appropriate physiologic assessment of the patient, and precise staging.

## Data Availability Statement

The raw data supporting the conclusions of this article will be made available by the authors, without undue reservation.

## Author Contributions

AA contributed to conception and design of the study, literature search using the MedHub database, extraction of data from the literature, and writing the first draft of the manuscript. JD, MB, and DR wrote sections of the manuscript. All authors contributed to manuscript revision, read, and approved the submitted version.

## Conflict of Interest

The authors declare that the research was conducted in the absence of any commercial or financial relationships that could be construed as a potential conflict of interest.

## Publisher’s Note

All claims expressed in this article are solely those of the authors and do not necessarily represent those of their affiliated organizations, or those of the publisher, the editors and the reviewers. Any product that may be evaluated in this article, or claim that may be made by its manufacturer, is not guaranteed or endorsed by the publisher.
